# Pneumococcal Colonization Rates in Patients Admitted to a United Kingdom Hospital with Lower Respiratory Tract Infection: a Prospective Case-Control Study

**DOI:** 10.1128/JCM.02008-15

**Published:** 2016-03-25

**Authors:** Andrea M. Collins, Catherine M. K. Johnstone, Jenna F. Gritzfeld, Antonia Banyard, Carole A. Hancock, Angela D. Wright, Laura Macfarlane, Daniela M. Ferreira, Stephen B. Gordon

**Affiliations:** a Respiratory Infection Group, Royal Liverpool and Broadgreen University Hospital Trust, Liverpool, United Kingdom; b Respiratory Infection Group, Liverpool School of Tropical Medicine, Liverpool, United Kingdom; c Local Comprehensive Research Network, Liverpool, United Kingdom

## Abstract

Current diagnostic tests are ineffective for identifying the etiological pathogen in hospitalized adults with lower respiratory tract infections (LRTIs). The association of pneumococcal colonization with disease has been suggested as a means to increase the diagnostic precision. We compared the pneumococcal colonization rates and the densities of nasal pneumococcal colonization by (i) classical culture and (ii) quantitative real-time PCR (qPCR) targeting *lytA* in patients with LRTIs admitted to a hospital in the United Kingdom and control patients. A total of 826 patients were screened for inclusion in this prospective case-control study. Of these, 38 patients were recruited, 19 with confirmed LRTIs and 19 controls with other diagnoses. Nasal wash (NW) samples were collected at the time of recruitment. Pneumococcal colonization was detected in 1 patient with LRTI and 3 controls (*P* = 0.6) by classical culture. By qPCR, pneumococcal colonization was detected in 10 LRTI patients and 8 controls (*P* = 0.5). Antibiotic usage prior to sampling was significantly higher in the LRTI group than in the control group (19 versus 3; *P* < 0.001). With a clinically relevant cutoff of >8,000 copies/ml on qPCR, pneumococcal colonization was found in 3 LRTI patients and 4 controls (*P* > 0.05). We conclude that neither the prevalence nor the density of nasal pneumococcal colonization (by culture and qPCR) can be used as a method of microbiological diagnosis in hospitalized adults with LRTI in the United Kingdom. A community-based study recruiting patients prior to antibiotic therapy may be a useful future step.

## INTRODUCTION

Recent studies suggest that detection and quantification of nasal pneumococci by quantitative real-time PCR (qPCR) targeting *lytA* might be used to identify the pneumococcus as the etiological pathogen in adults with pneumonia (1) and might be useful as a disease severity marker (2). In that study, South African patients with community-acquired pneumonia (CAP) were more frequently colonized than controls by classical culture (44.9 versus 11.7%) and qPCR (62.8 versus 19.8%), and, in addition, patients with pneumococcal CAP were also noted to have higher colonization densities than asymptomatic controls (1). By applying a cutoff of 8,000 copies/ml to the qPCR data, Albrich et al. (1) found that 52.5% of patients were considered to have pneumococcal CAP compared with 27.1% diagnosed using standard tests.

The association of pneumonia and pneumococcal colonization has been previously noted in children, in whom those with radiologically confirmed pneumonia were more frequently colonized with pneumococci than those without (3) and had higher-density colonization rates than those with bronchitis or without disease (4). In contrast, in elderly individuals, very low colonization rates have been shown: 0.3% in pneumococcal vaccine-naive hospitalized Australians (by classical culture), of whom 10 had respiratory infections (5), and 2.3% in a Portuguese community cohort (6). In developed countries, pneumococcal colonization rates in healthy adults are between 1 and 18% and are affected by age, immune status, antibiotic use, household composition, and contact with children (7, 8). There are no published data on pneumococcal colonization rates in hospitalized patients with respiratory infection in the United Kingdom.

We therefore aimed to determine the rates and densities of pneumococcal colonization by (i) classical culture and (ii) qPCR in hospitalized adult patients with lower respiratory tract infection (LRTI) compared with those of age- and gender-matched controls in a developed country setting.

## MATERIALS AND METHODS

### Screening and recruitment.

We recruited hospitalized adults with LRTI at the Royal Liverpool and Broadgreen University Hospital from January to July 2013 within 72 h of admission. The syndrome of LRTI was defined as symptoms of respiratory infection with clinical signs with or without radiological consolidation, which therefore meet a British Thoracic Society (BTS) definition of pneumonia as used in community (general practitioner [GP]) practice. The clinical signs of LRTI included ≥2 of the following: cough, breathlessness, pleuritic chest pain, fever, and increased or new sputum production. The exclusion criteria were the following: infective or noninfective exacerbations of chronic obstructive pulmonary disease (IECOPD), asthma or bronchiectasis (without radiological consolidation), aspiration pneumonia, oxygen saturations of <86% on air, suspected tuberculosis (TB), and neutropenia. Patients with IECOPD were excluded as their exacerbations are known to commonly be due to viruses (up to 60%) (9, 10), Haemophilus influenzae (up to 33%) (11), and Moraxella catarrhalis (around 10%) (12) rather than to pneumococci. Patients who had been hospital inpatients for ≥72 h or had recently been discharged from the hospital (≤14 days before) were excluded since it is likely that their nasal flora would have been altered due to hospital exposures. Patients with oxygen saturations of <86% on air were excluded since it was felt unsafe to remove their oxygen in order to perform a nasal wash (NW).

A carefully selected control group of hospitalized patients with no signs of respiratory infection were recruited within 7 days (where possible or as soon after as possible) of the LRTI patients. The control group patients were matched for age (within 10 years of those of the LRTI patients) and gender. The exclusion criteria were the following: oxygen saturation of <86% on air, neutropenia, time after admission of ≥7 days, and recent hospital discharge of ≤14 days.

The study team members were in regular communication with the hospital capacity team, the ward-based case managers, and the nursing and medical coordinators in the accident and emergency department, the acute medical admissions unit (AMAU), and the respiratory wards from Monday to Thursday. Through regular education, information dissemination events, and daily interactions with the study team, key staff were made aware of the study and its aims, objectives, and potential participants. A list of potential participants was generated on a daily basis in combination with these personnel. To recruit LRTI patients, we targeted screening to the AMAU and the respiratory and infectious disease wards; for control participants, we targeted the surgical wards.

Patient eligibility was confirmed by a review of the medical records; with permission of the attending team, patient consent was sought prior to recruitment. Baseline clinical data on age, gender, history of the presenting complaint, past medical history, vaccination history, antibiotic prescription, and contact with children (defined as at least alternate-day contact with children aged ≤10 years) were recorded. NW and urine samples were collected within 12 h of recruitment. The study was approved by the North West−Liverpool East NHS Research Ethics Committee (12/NW/0713) and was registered at ClinicalTrials.gov under registration no. NCT01861184.

### Sampling.

NW samples was collected on the day of recruitment with a maximum of 20 ml of normal saline instilled into the nasopharynx as previously described (13, 14). A minimum of 5 ml of normal saline was recovered and processed in all cases. Briefly, samples were immediately centrifuged at high speed (3,345 × *g*) to obtain a bacterial pellet (14). The pellets were resuspended in 100 μl of skimmed milk-tryptone-glucose-glycerol (STGG) medium, and the total volume of the suspension was determined. Samples were then serially diluted on blood agar, and the CFU/ml of the NW samples were determined on the next day. In a subset of samples that exceeded 7 ml, a proportion of the sample (3 to 5 ml) was removed and centrifuged at 836 × *g* to obtain cellular material after which the supernatant was readded to the rest of the sample for the high-speed spin.

### DNA extraction and qPCR.

DNA was extracted from 200 μl of the NW bacterial pellet stored in STGG using the QIAamp DNA minikit and the Centers for Disease Control and Prevention protocol (15). Briefly, the pellet was resuspended in 200 μl of Tris-EDTA (TE) buffer containing 0.04 g/ml lysozyme and 75 U/ml mutanolysin (Sigma) and incubated at 37°C for 1 h. Following incubation, 20 μl of proteinase K and 200 μl of buffer AL were added to the sample, which was vortexed and then incubated at 56°C for 30 min. The sample was then centrifuged briefly, and 260 μl of ethanol was added. All subsequent steps followed the manufacturer's instructions. DNA was eluted in 100 μl of Qiagen elution buffer and stored at −20°C.

The colonization density was determined by targeting the pneumococcal autolysin *lytA* gene (16). A no-template control, a negative-extraction control (parallel extraction of TE buffer), and a Streptococcus pneumoniae (BHN418)-positive control were included in each run. DNA was amplified with the Mx3005P system (Stratagene), and data were analyzed using the instrument's software. A sample was considered positive if both duplicates had a mean cycle threshold (*C_T_*) value of <35. Values of >8,000 copies/ml were considered clinically relevant according to Albrich and colleagues (1).

### BinaxNOW.

An immunochromatographic membrane test (ICT) (BinaxNOW Streptococcus pneumoniae; Binax) was performed on all patients' unconcentrated urine specimens, according to the manufacturer's recommendations.

## RESULTS

### Screening and recruitment.

**FIG 1:**
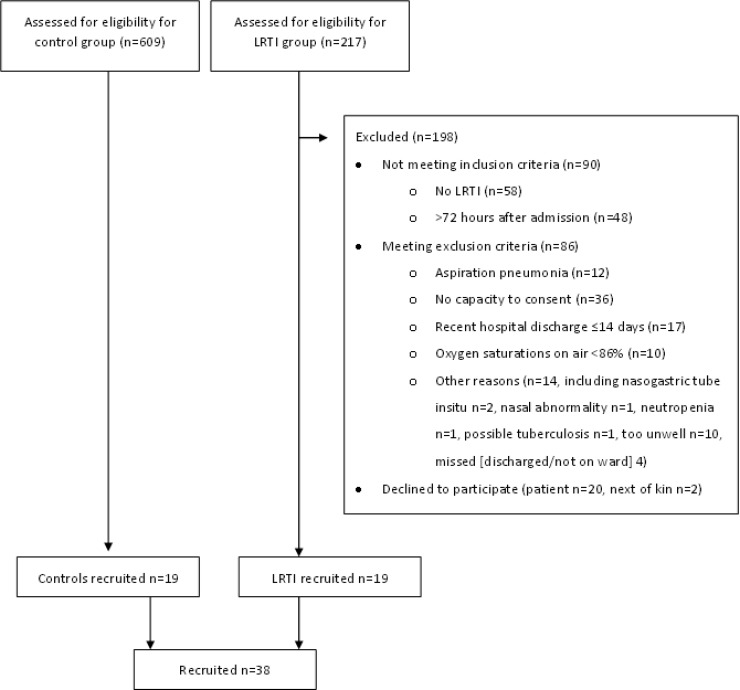
Screening and recruitment flowchart. Reasons for nonrecruitment for lower respiratory tract infection (LRTI) patients are detailed. The total number of patients screened was 826. Note that multiple reasons for nonrecruitment per patient were possible.

We screened 826 patients and recruited 19 LRTI and 19 control (age-, gender-, and season-matched) patients. Of the 217 “potential” LRTI patients, 198 were not eligible (Fig. 1). Fifty-eight patients did not have an LRTI syndrome (acute exacerbation [AE] chronic obstructive pulmonary disease [COPD], *n* = 22; AE bronchiectasis, *n* = 5; AE asthma, *n* = 3; AE pulmonary fibrosis, *n* = 1; and alternative diagnoses, including pulmonary embolus [PE], congestive cardiac failure [CCF], sepsis of unknown cause, and adult acute respiratory distress syndrome [ARDS], *n* = 30), 36 patients did not have the capacity to consent (predominately due to dementia or acute delirium), 48 patients were identified at >72 h after admission and 17 after a recent hospital discharge of ≤14 days before, 20 patients declined to participate and 2 next of kin refused permission for their relatives to participate, 10 patients had oxygen saturations of <86% on air, 12 patients had aspiration pneumonia, and 14 patients were excluded for other reasons.

We planned to recruit 100 patients for each arm of the study, but stopped recruiting on the grounds of futility after an interim analysis noted 100% antibiotic usage prior to recruitment in the LRTI group and low rates of colonization (on culture) and a high screening failure rate (778/816, 95.4%). Recruiting age-matched controls was difficult, especially for the younger LRTI patients (aged 36 to 46 years). In 9 cases, the time between the recruitment of the LRTI patient and the control was >7 days (range, 9 to 43 days). We know from our Experimental Human Pneumococcal Colonization (EHPC) studies that antibiotic usage terminates pneumococcal colonization, meaning that continued recruitment in this population was unethical.

### Sampling. (i) Rate of colonization.

All patients successfully provided a nasal sample. One patient was unable to replicate the NW technique (according to the protocol) and therefore had a nasopharyngeal swab instead. NW volumes were not significantly different between LRTI patients and controls (Table 1). Pneumococcal colonization was detected using classical microbiology cultures in 1 LRTI patient and 3 controls (*P* = 0.6). With qPCR, 10 LRTI patients and 8 controls were positive (*P* = 0.516) (Table 2). One of the controls was positive for colonization by culture but was considered negative by qPCR as the *C_T_* value was >35.

**TABLE 1:** Baseline demographics, antibiotic status, nasal wash volume returned, and evidence of pneumococcal disease investigation results for patients with lower respiratory tract infection and age- and gender-matched hospitalized controls

Parameter	Results for:		*P*
	LRTI^*a*^ patients (*n* = 19)	Controls(*n* = 19)	
Male gender (n [%])	9 (47.4)	9 (47.4)	1.000^*b*^
Age (mean ± SD) (yr)	64.47 ± 15.78	64.58 ± 14.50	0.954^*c*^
Smoker/ex-smoker (n [%])	15 (78.9)	10 (52.6)	0.170^*d*^
23PPV^*e*^ (n [%])	7 (36.8)	8 (42.1)	0.740^*b*^
Contact with children (n [%])	10 (52.6)	12 (63.2)	0.511^*b*^
Antibiotics at time of recruitment (n [%])	19 (100)	3 (15.8)	0.0001^*d*^
Nasal wash vol returned (mean ± SD) (ml)	10.14 ± 3.14	10.36 ± 4.83	0.855^*c*^
Evidence of pneumococcal disease: BinaxNOW urine test positive (n [%])	2 (10.5)	0 (0)	0.486^*d*^
Evidence of pneumococcal disease: blood or sputum culture positive (n [%])	0 (0)	NA^*f*^	NA

a LRTI, lower respiratory tract infection.

b Chi-square test.

c Mann-Whitney U test.

d Fisher's exact test.

e 23PPV, 23-valent pneumococcal polysaccharide vaccine (Pneumovax).

f NA, not applicable.

**TABLE 2:** Pneumococcus identification (by culture and qPCR) and density (by qPCR) in patients with lower respiratory tract infection and age- and gender-matched hospitalized controls^*a*^

Parameter	Results for:		*P*
	LRTI patients (*n* = 19)	Controls (*n* = 19)	
Culture positive (n [%])	1 (5)	3 (15.8)	0.604^*b*^
*q*PCR^*c*^ positive at detection limit (n [%])	10 (52.6)	8 (42.1)	0.516^*d*^
Density (by qPCR) (geometric mean copies/ml [95% CI^*e*^])	3,066 (1,225–7,675)	2,208 (244–19,972)	0.408^*f*^
Clinically relevant density (by qPCR) of >8,000 copies/ml	3	4	0.999^*b*^

a Note the low rates of culture positivity and high rates of qPCR positivity in both the lower respiratory tract infection (LRTI) and control groups.

b Fisher's exact test.

c qPCR, quantitative PCR.

d Chi-square test.

e CI, confidence interval.

f Mann-Whitney U test.

### (ii) Density of colonization by qPCR.

For qPCR, a cutoff value of >8,000 copies/ml was used to define the clinical relevance (1). In our study, 3 LRTI patients and 4 controls had values of >8,000 copies/ml. Of the 3 LRTI patients, only 1 was culture positive; of the 4 controls, 2 were culture positive (Table 2). Of the 4 patients overall who were culture positive, 3 had >8,000 copies/ml (1 in the LRTI group and 2 in the control group).

### Clinical data.

Antibiotic usage prior to sampling was significantly higher in the LRTI patients than in the controls (19 versus 3; *P* < 0.001). Radiological consolidation was present in 7 out of 19 LRTI patients; only 2 out of 38 urine samples were positive using BinaxNOW. None of the LRTI patients recruited were pneumococcal sputum or blood culture positive. There were no statistical differences between the groups with regard to smoking, contact with children of age <10 years, or 23-valent pneumococcal polysaccharide vaccine (23PPV) (Pneumovax) vaccination (Table 1).

## DISCUSSION

The anticipated high rate of pneumococcal colonization (by culture with or without qPCR) in the LRTI group was not found, given that the antibiotic usage (preadmission/prerecruitment) was significantly different between the LRTI and control groups, with all LRTI patients having received at least 2 doses prior to NW sampling; this is likely to have resulted in culture negativity. However, we also found no significant differences in the colonization rates using qPCR and colonization density between the LRTI and control groups. There were also no significant differences in the colonization rates in polysaccharide-vaccinated (23PPV, Pneumovax) and unvaccinated patients, consistent with previous literature reports stating that the vaccine does not protect against colonization (17, 18).

A large number of patients were referred as potential LRTI patients. Alternative diagnoses such as PE, CCF, noninfective exacerbation of pulmonary fibrosis, sepsis of unknown cause, and aspiration pneumonia were common. This diagnostic imprecision has important implications for the use of NW sampling as a diagnostic technique since it would lead to many inappropriate samples being collected. We have previously demonstrated that confusion is common in LRTI patients (>20%) (19). The rates of LRTI increase with age (63% of the patients admitted with CAP were aged >65 years and 25% were ≥85 years old) (20) as do the rates of comorbidities (including dementia); therefore, recent hospital admission is also common.

The main strength of this study is the large number of screened patients; the LRTI patients were well phenotyped, and the controls were matched in age, gender, and time of recruitment and had similar smoking habits, 23PPV (Pneumovax) vaccination rates, and child contact. Our cohort did not have CAP by the strict definition of radiological consolidation; rather a broad study group of LRTI patients was chosen due to its clinical relevance in United Kingdom hospital practice and admissions, making these results very generalizable. Nationally, the antibiotic prescribing rate by GPs for LRTIs is very high but is lower for clinically diagnosed CAP (due to the usual immediate hospitalization) (21).

Accurately diagnosing pneumonia is challenging; interdoctor variability in reporting of radiologically confirmed pneumonia is common (22). Studies of patients who have pneumonia diagnosed radiologically as an inclusion criterion may be less applicable to everyday hospital medicine. LRTI may be a more useful term for this clinical syndrome, particularly in instances where the guidelines suggest clinical rather than radiological diagnosis (20). Liverpool is in northwest England and has the second highest LRTI rate (age standardized episodes/1,000 person years) and the third highest CAP rate nationally (21). It is therefore an ideal area for recruitment for respiratory infection studies, although community antibiotic prescription rates are high. The Royal Liverpool hospital has ∼1,400 admissions per year that are coded as “pneumonia”; approximately 20% of these cases are not community acquired or the patients have no radiological features of pneumonia.

The limitations of the study are that this is a single center study, which may reduce the generalizability of the results, specifically in areas where community antibiotic prescription rates are lower, that we were unable to fully recruit for the study despite the high numbers of individuals screened, and that the NW sampling technique, rather than nasopharyngeal swab, for pneumococcal isolation may not have been ideal in this elderly population, since the research nurses noted poor performance and lower yields than in the cohort of healthy volunteers in which we commonly use this technique (data not shown). Nevertheless, patient comfort is higher (23), and the sensitivity for colonization density is very high (24). We know from our Experimental Human Pneumococcal Colonization (EHPC) studies that antibiotic usage terminates pneumococcal colonization; after an interim analysis noted 100% antibiotic usage in the LRTI group prior to recruitment and low rates of colonization (on culture), the study was stopped as continued recruitment in this population was unethical.

Previous studies have shown colonization rates of 44.9% and 62.8% in patients with radiologically confirmed CAP compared to 11.7% and 19.8% in controls by culture and qPCR, respectively (1); in comparison, we detected colonization rates of 5% and 15.8% (>8,000 copies/ml) in patients with LRTI and 15.8% and 21.0% (>8,000 copies/ml) in controls. We therefore noted high rates of PCR positivity in both groups and low rates of culture positivity in our LRTI patients compared with those for the CAP patients in the previous study. The differences between the two studies may be due to the fact that our patient cohort was considerably older (64.5 versus 38.4 years old) (1), had low rates of radiologically confirmed pneumonia (36.8%), had high rates of prior antibiotic treatment, had high rates of contact with children, and were presumed to be HIV uninfected (the overall incidence of HIV infection is low in Liverpool: 15/100,000, with a prevalence of 0.17% in 2011 [D. Sloan, unpublished local data]). Previously in Liverpool, we found natural colonization rates of 10% in healthy nonsmoking volunteers by classical culture (25/249, aged 23 years old [standard deviation, ±5.7]) (unpublished data). The higher rate (15.8%) in this cohort may be related to the high rates of contact with children and smoking history or our patients.

qPCR can deliver results within a few hours (usually 3 to 6 h), which might impact the critical phase of early clinical care (25); however, it does not distinguish between viable (live) and nonviable (dead) bacteria or determine whether the bacteria are pathogens or colonizers (26, 27). Specificity can also be an issue with qPCR, and there have been concerns that *lytA* may not discriminate between S. pneumoniae and Streptococcus *viridans*; however, *lytA* is currently the most widely used target gene for pneumococci, and we have previously shown that our assay specificity (24) is in line with that reported by others (16).

Within this cohort, all LRTI patients had taken antibiotics prior to sampling, which probably accounts for the higher positivity rate of qPCR over culture. Prior antibiotic treatment can lower plasma and pleural fluid PCR loads (28) as well as sputum and blood culture positivity. It is not known how rapidly pneumococci will be undetectable by qPCR in the NW samples of those with previous pneumococcal colonization after antibiotic therapy.

Albrich and colleagues suggested that a density of 10^3^ to 10^4^ may be the critical value at which colonization leads to infection (1); however, we have found densities as high as or higher than these in our cohort of healthy volunteers after experimental colonization without infection (24, 29). Colonization densities were not different in the LRTI and control groups; we also found high mean densities of ≥10^3^ in those without infection (*n* = 4 controls). It is possible, therefore, that if colonization is dense and in the setting of the correct clinical syndrome, then the pneumococcus is a likely pathogen. Again, an important difference between the two study groups may be HIV infection status. Only 10.5% (2/19) of our LRTI group were BinaxNOW positive compared to 72.7% in patients with nonbacteremic CAP in another study (1). The BinaxNOW results remain positive for at least 7 days after the initiation of antibiotic treatment (30); notably, our urine samples were taken up to 72 h after admission but often several days after antibiotics had been started. Previous antibiotic therapy has been noted to decrease culture and qPCR positivity by up to 50% (1).

In conclusion, we have shown that pneumococcal colonization (assessed by culture and qPCR) cannot be used as a method of diagnosis for pneumococcal blood culture-negative hospitalized adults with LRTI in the United Kingdom, since such patients have already received antibiotic therapy in the community setting and the laboratory test is nondiscriminatory. Further, the number of adults tested for potential LRTI on screening would be impracticable in terms of staff resources. A community-based study recruiting patients prior to antibiotic therapy may, however, be a useful future step.
